# Supplementary material for: INPP4B promotes PI3Kα-dependent late endosome formation and Wnt/β-catenin signaling in breast cancer
Source: Nat Commun. 2021 May 25;12:3140. doi: 10.1038/s41467-021-23241-6 (PMC8149851; doi:10.1038/s41467-021-23241-6)
Supplement: Supplementary file 1 — Supplementary Information [file 41467_2021_23241_MOESM1_ESM.pdf]

Supplementary Information

INPP4B promotes PI3Kα-dependent late endosome formation and Wnt/β-catenin signaling in breast cancer

Samuel J. Rodgers, Lisa M. Ooms, Viola M.J. Oorschot, Ralf B. Schittenhelm, Elizabeth V. Nguyen, Sabryn A. Hamila, Natalie Rynkiewicz, Rajendra Gurung, Matthew J. Eramo, Absorn Sriratana, Clare G. Fedele, Franco Caramia, Sherene Loi, Genevieve Kerr, Helen E. Abud, Georg Ramm, Antonella Papa, Andrew M. Ellisdon, Roger J. Daly, Catriona A. McLean, Christina A. Mitchell.

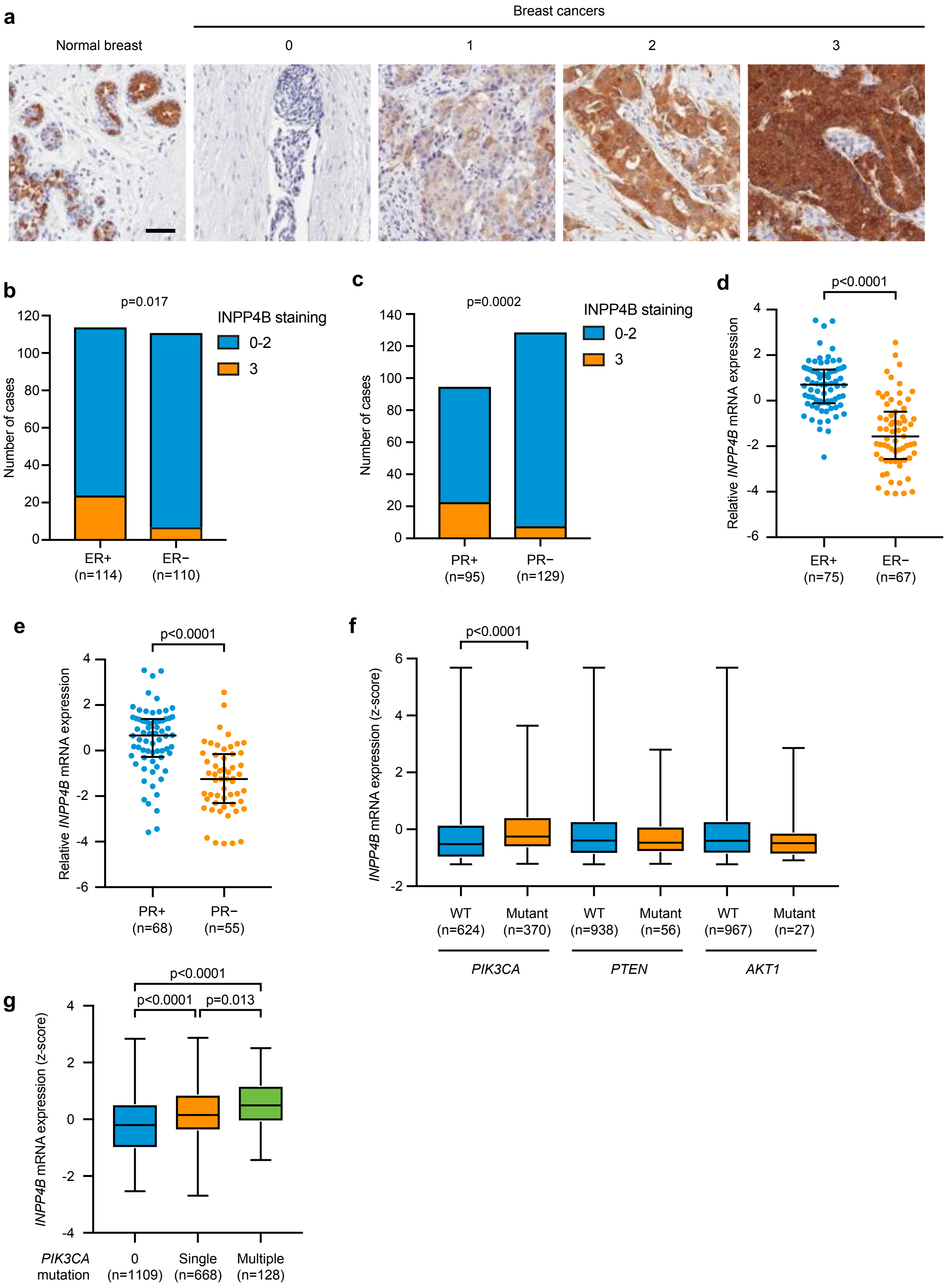
a
Breast cancers
Normal breast
0
1
2
3
b
p=0.017
INPP4B staining
0-2
3
Number of cases
ER+ (n=114)
ER− (n=110)
c
p=0.0002
INPP4B staining
0-2
3
Number of cases
PR+ (n=95)
PR− (n=129)
d
p<0.0001
Relative INPP4B mRNA expression
ER+ (n=75)
ER− (n=67)
e
p<0.0001
Relative INPP4B mRNA expression
PR+ (n=68)
PR− (n=55)
f
p<0.0001
INPP4B mRNA expression (z-score)
WT (n=624)
Mutant (n=370)
WT (n=938)
Mutant (n=56)
WT (n=967)
Mutant (n=27)
PIK3CA
PTEN
AKT1
g
p<0.0001
p<0.0001
p=0.013
INPP4B mRNA expression (z-score)
PIK3CA mutation
0 (n=1109)
Single (n=668)
Multiple (n=128)

Supplementary Figure 1: High INPP4B expression is associated with ER-positive and PR-positive breast cancer.

(a) Representative images of high (3), moderate (2), low (1) or no (0) INPP4B staining from tissue microarrays (n=224 cases).

(b, c) High INPP4B protein expression was correlated with ER+ **(b)** or PR+ **(c)** breast cancers from tissue microarrays.

(d, e) *INPP4B* mRNA expression was correlated with ER+ **(d)** or PR+ **(e)** breast cancers from Tissue Scan Breast Cancer cDNA Arrays I-IV (OriGene). Data represent median *INPP4B* mRNA expression ± 25th and 75th percentiles

(f) *INPP4B* mRNA expression was stratified by *PIK3CA*, *PTEN* or *AKT1* mutation status in breast tumors from the TCGA cohort (n=994 cases). The centre line indicates the median, the lower bound of the box indicates the 25th percentile, the upper bound of the box represent the 75th percentile, the lower whisker extends from the 25th percentile to the minimum value, and the upper whisker extends from the 75th percentile to the maximum value.

(g) *INPP4B* mRNA expression was stratified by the number of *PIK3CA* mutations in breast tumors from the METABRIC cohort (n=1904 cases). The centre line indicates the median, the upper and lower bounds of box indicate the 25th and 75th percentiles of the data, and the upper and lower whiskers represent minimum to maximum values.

Scale bar 100 μM **(a)**.

p values determined by Fisher's exact test are indicated in **(b**, **c)**, by two-tailed unpaired Mann-Whitney test in **(d-f)**, and by Kruskal-Wallis test with Dunn's post hoc test in **(g)**.

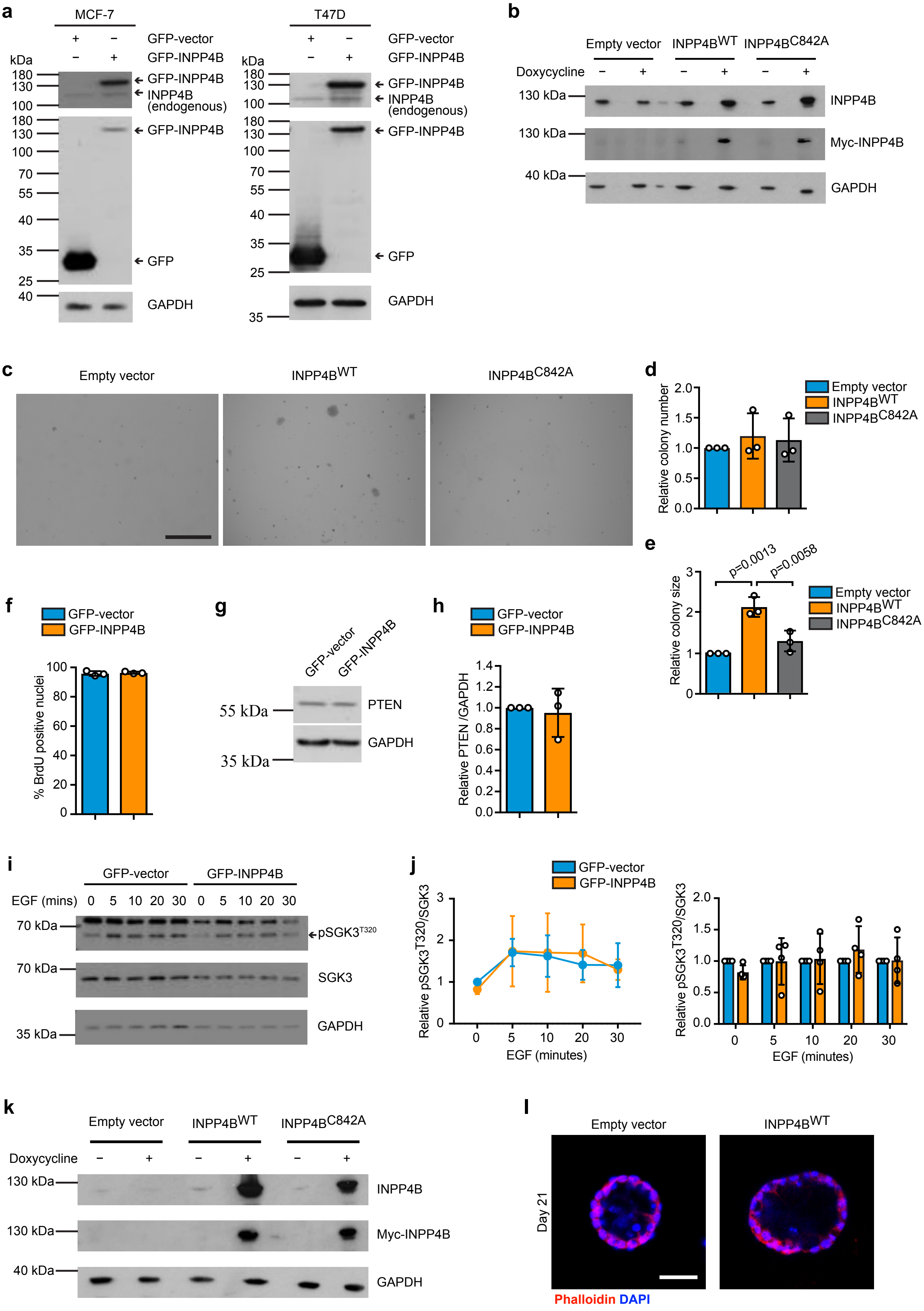

a
MCF-7
T47D
GFP-vector
GFP-INPP4B
kDa
180
130
100
70
55
40
35
25
GFP-INPP4B
INPP4B (endogenous)
GFP
GAPDH
b
Empty vector
INPP4BWT
INPP4BC842A
Doxycycline
130 kDa
40 kDa
INPP4B
Myc-INPP4B
GAPDH
c
Empty vector
INPP4BWT
INPP4BC842A
d
Relative colony number
2.0
1.5
1.0
0.5
0.0
Empty vector
INPP4BWT
INPP4BC842A
e
Relative colony size
p=0.0013
p=0.0058
3
2
1
0
f
GFP-vector
GFP-INPP4B
% BrdU positive nuclei
100
80
60
40
20
0
g
GFP-vector
GFP-INPP4B
55 kDa
35 kDa
PTEN
GAPDH
h
Relative PTEN /GAPDH
1.4
1.2
1.0
0.8
0.6
0.4
0.2
0.0
i
GFP-vector
GFP-INPP4B
EGF (mins)
0 5 10 20 30 0 5 10 20 30
70 kDa
35 kDa
pSGK3T320
SGK3
GAPDH
j
Relative pSGK3T320/SGK3
EGF (minutes)
0
5
10
20
30
k
Empty vector
INPP4BWT
INPP4BC842A
Doxycycline
130 kDa
40 kDa
INPP4B
Myc-INPP4B
GAPDH
l
Empty vector
INPP4BWT
Day 21
Phalloidin DAPI

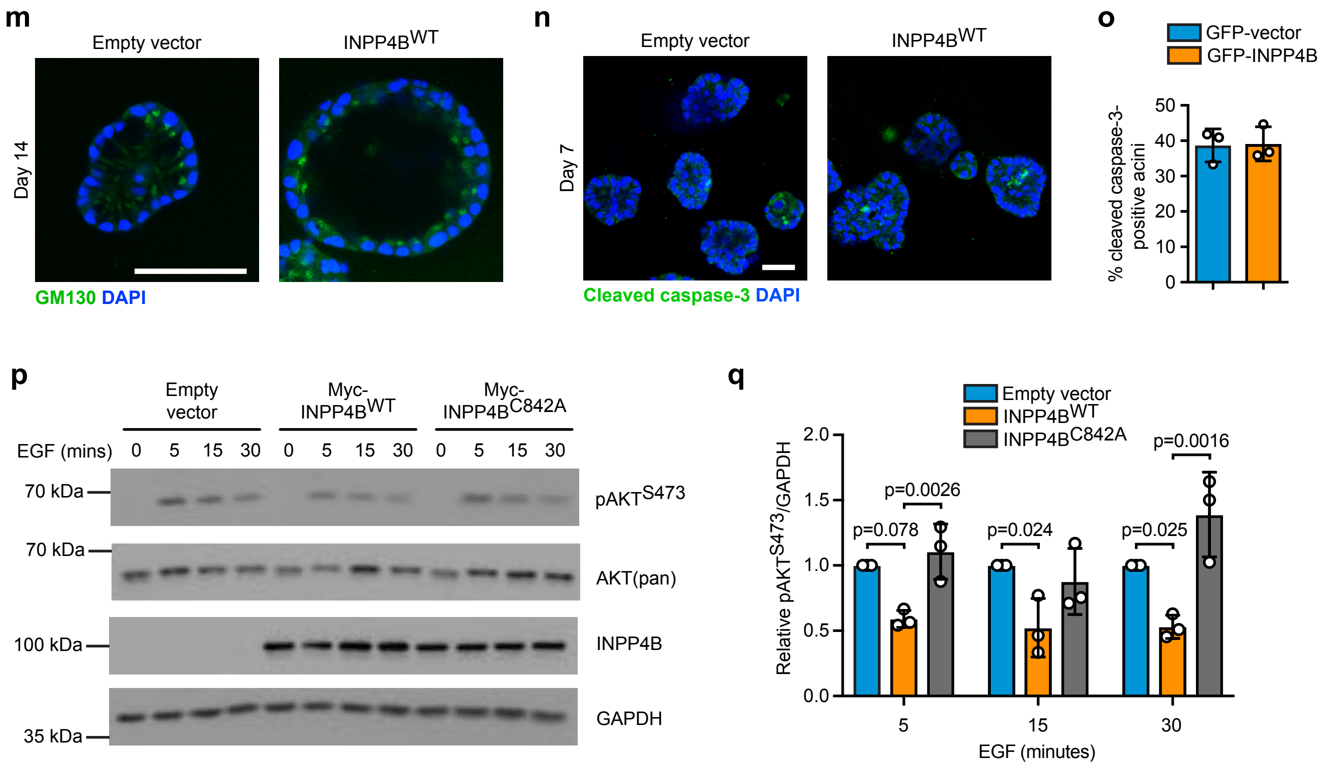

Supplementary Figure 2: Generation of stable INPP4B overexpressing breast cancer and mammary epithelial cells.

(a) MCF-7 or T47D cells were transduced with lentiviral particles encoding GFP-INPP4B or GFP-vector. Cells were lysed, and immunoblotted with GFP or INPP4B antibodies, or GAPDH antibodies as a loading control.

(b) MCF-7 cells were transduced with lentiviral particles encoding doxycycline-inducible wild-type INPP4B^{WT}, catalytically inactive INPP4B^{C842A} or empty vector, and co-transduced with particles encoding the Tet3G transactivator protein. Cells were cultured in growth media ± doxycycline (1 µg/mL) for 24 hours, then lysed and immunoblotted with INPP4B or Myc-tag antibodies, or GAPDH antibodies as a loading control.

(c-e) MCF-7 cells expressing Myc-INPP4B^{WT}, catalytically inactive Myc-INPP4B^{C842A} or empty vector were suspended in 0.3% soft agar and cultured for 4 weeks to allow anchorage-independent cell growth **(c)**. Data represent relative number of colonies **(d)** and relative colony size (n>50 colonies/experiment) **(e)** ± SD (n=3 experiments in triplicate).

(f) MCF-7 cells expressing GFP-INPP4B or GFP-vector were grown in serum-containing media for 24 hours, incubated with BrdU overnight then fixed and immunostained with BrdU antibodies and DAPI. Data represent mean percentage of BrdU-positive cells ± SD (n=3 experiments, >300 cells/experiment).

(g, h) MCF-7 cells expressing GFP-INPP4B or GFP-vector were lysed and immunoblotted with PTEN antibodies or GAPDH antibodies as a loading control **(g)**. Data represent the mean PTEN levels relative to GAPDH ± SD (n=3 experiments) **(h)**.

(i, j) MCF-7 cells expressing GFP-INPP4B or GFP-vector were serum-starved overnight, then stimulated with EGF (100 ng/mL) for the indicated times. Cells were lysed and immunoblotted with $pSGK3^{T320}$ or SGK3 antibodies, or GAPDH antibodies as a loading control **(i)**. Data represent the mean relative $pSGK3^{T320}$ levels relative to SGK3 ± SD (n=4 experiments) **(j)**.
(k) MCF-10A cell were transduced with lentiviral particles containing plasmids encoding Myc-$INPP4B^{WT}$, catalytically inactive Myc-$INPP4B^{C842A}$ or empty vector under a doxycycline-inducible promoter, and co-transduced with particles encoding the Tet3G transactivator protein. Cells were cultured in growth media ± doxycycline (1 µg/mL) for 24 hours, then lysed and immunoblotted with INPP4B or Myc-tag antibodies, or GAPDH antibodies as a loading control.
(l-o) MCF-10A acini expressing Myc-$INPP4B^{WT}$ or empty vector were cultured in Matrigel for the indicated number of days, then fixed and stained with phalloidin **(l)**, GM130 **(m)** or cleaved caspase-3 **(n)** antibodies and co-stained with DAPI. Data represent the mean percentage of acini with cleaved-caspase 3 staining ± SD (n=3 experiments, >50 acini/experiment) **(o)**.
(p, q) MCF-10A cells expressing $INPP4B^{WT}$, $INPP4B^{C842A}$ or empty vector were serum-starved overnight, then stimulated with EGF (100 ng/mL) for the indicated times. Cells were lysed and immunoblotted with $pAKT^{S473}$ or AKT(pan) antibodies, or GAPDH antibodies as a loading control **(p)**. Data represent the mean relative $pAKT^{S473}$ levels relative to GAPDH ± SD (n=3 experiments) **(q)**.
Scale bar 50 µm **(l-n)**, 1 mm **(c)**.
p values determined by one-way ANOVA with Tukey post hoc test are indicated in **(e)**, or by one-way ANOVA in **(q)**.

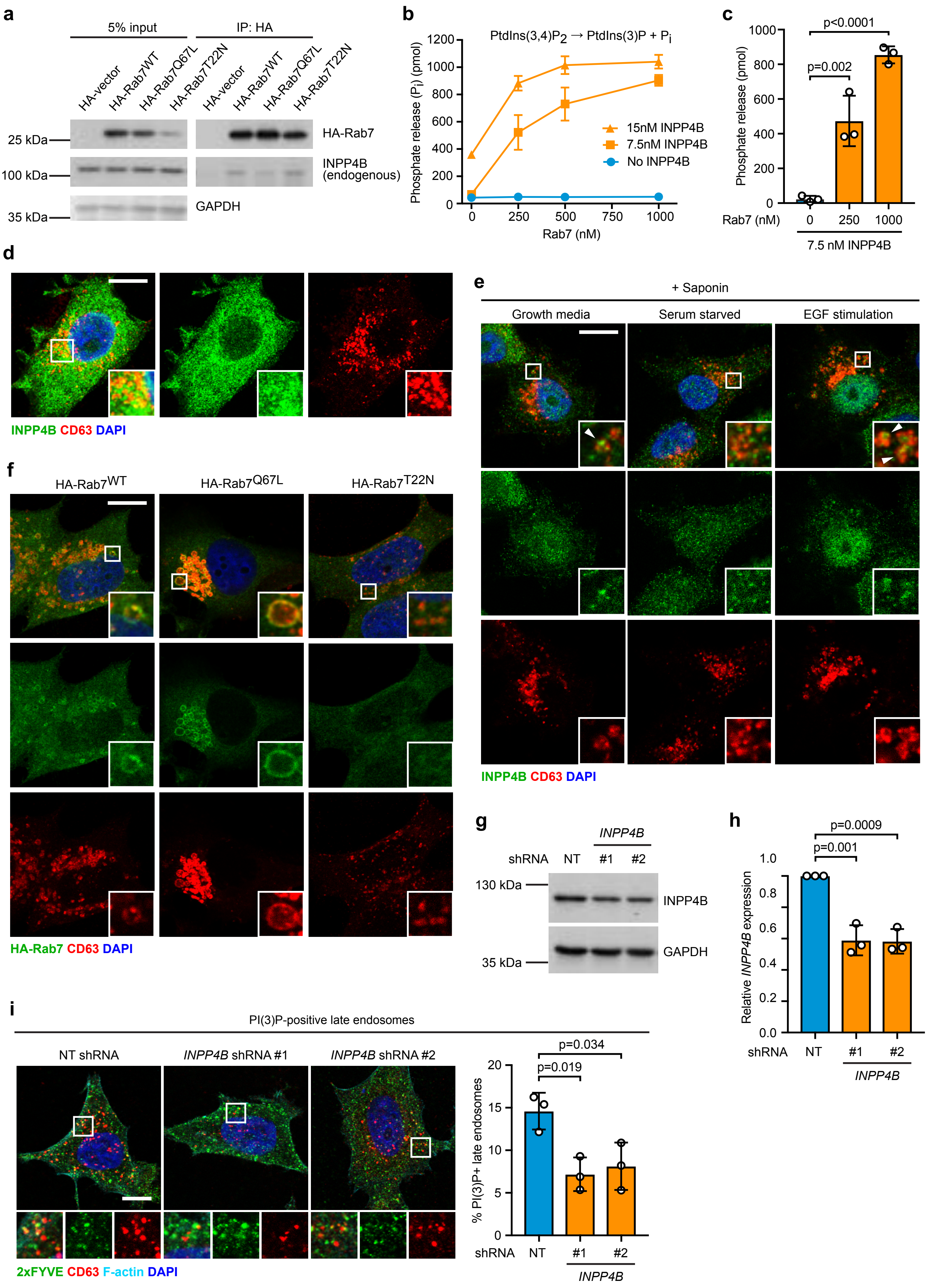
a
5% input
IP: HA
HA-vector
HA-Rab7WT
HA-Rab7Q67L
HA-Rab7T22N
25 kDa
100 kDa
35 kDa
HA-Rab7
INPP4B (endogenous)
GAPDH
b
PtdIns(3,4)P2 → PtdIns(3)P + Pi
Phosphate release (Pi) (pmol)
Rab7 (nM)
15nM INPP4B
7.5nM INPP4B
No INPP4B
c
p<0.0001
p=0.002
Phosphate release (pmol)
Rab7 (nM)
7.5 nM INPP4B
d
INPP4B CD63 DAPI
e
+ Saponin
Growth media
Serum starved
EGF stimulation
INPP4B CD63 DAPI
f
HA-Rab7WT
HA-Rab7Q67L
HA-Rab7T22N
HA-Rab7 CD63 DAPI
g
INPP4B
shRNA
NT
#1
#2
130 kDa
35 kDa
INPP4B
GAPDH
h
p=0.0009
p=0.001
Relative INPP4B expression
shRNA
NT
#1
#2
INPP4B
i
PI(3)P-positive late endosomes
NT shRNA
INPP4B shRNA #1
INPP4B shRNA #2
2xFYVE CD63 F-actin DAPI
p=0.034
p=0.019
% PI(3)P+ late endosomes
shRNA
NT
#1
#2
INPP4B

Supplementary Figure 3: INPP4B regulates PI(3)P generation on late endosomes.

(a) MCF-7 cells expressing HA-Rab7^{WT}, HA-Rab7^{Q67L}, HA-Rab7^{T22N} or HA-vector were lysed and subjected to immunoprecipitation using HA antibodies. Bound fractions and soluble lysates (5% of input) were subjected to immunoblotting to detect endogenous INPP4B.

(b, c) Recombinant purified INPP4B PI(3,4)P_2 4-phosphatase activity was measured in the presence of recombinant purified Rab7 using malachite green assays. Data represent phosphate release **(b)** and background corrected phosphate release following 20 minute reactions **(c)** ± SD (n=3 experiments).

(d) MCF-7 cells were fixed and immunostained using INPP4B and CD63 antibodies, and DAPI.

(e) MCF-7 cells were cultured in growth media, serum starved overnight or serum-starved overnight then stimulated with EGF (100 ng/mL) for 15 minutes. Cells were treated with saponin (0.02% w/v) to remove cytoplasmic proteins, then fixed and immunostained using INPP4B and CD63 antibodies, and co-stained with DAPI.

(f) MCF-7 cells expressing HA-Rab7^{WT}, HA-Rab7^{Q67L} or Rab7^{T22N} were fixed and immunostained using HA and CD63 antibodies, and co-stained with DAPI.

(g, h) MCF-7 cells were transduced with lentiviral particles encoding NT, *INPP4B* #1 or *INPP4B* #2 shRNA. Cells were lysed and subjected to immunoblotting with INPP4B antibodies or GAPDH antibodies as a loading control **(g)**. Data represent the relative expression of INPP4B relative to GAPDH expression ± SD (n=3 experiments) **(h)**.

(i) MCF-7 cells expressing NT, *INPP4B* #1 or *INPP4B* #2 shRNA were fixed and immunostained using recombinant purified GST-2xFYVE and CD63 antibodies, and co-stained with DAPI and phalloidin. Data represent the percentage of 2xFYVE+ late endosomes ± SD (n=3 experiments, >30 cells/experiment).

The inset panels at the lower right of or beneath each image are higher power regions of the boxed areas. Scale bar is 10 μm **(d-f)**, **(i)**.

p values determined by one-way ANOVA with Tukey post hoc test are indicated in **(c**, **h**, **i)**.

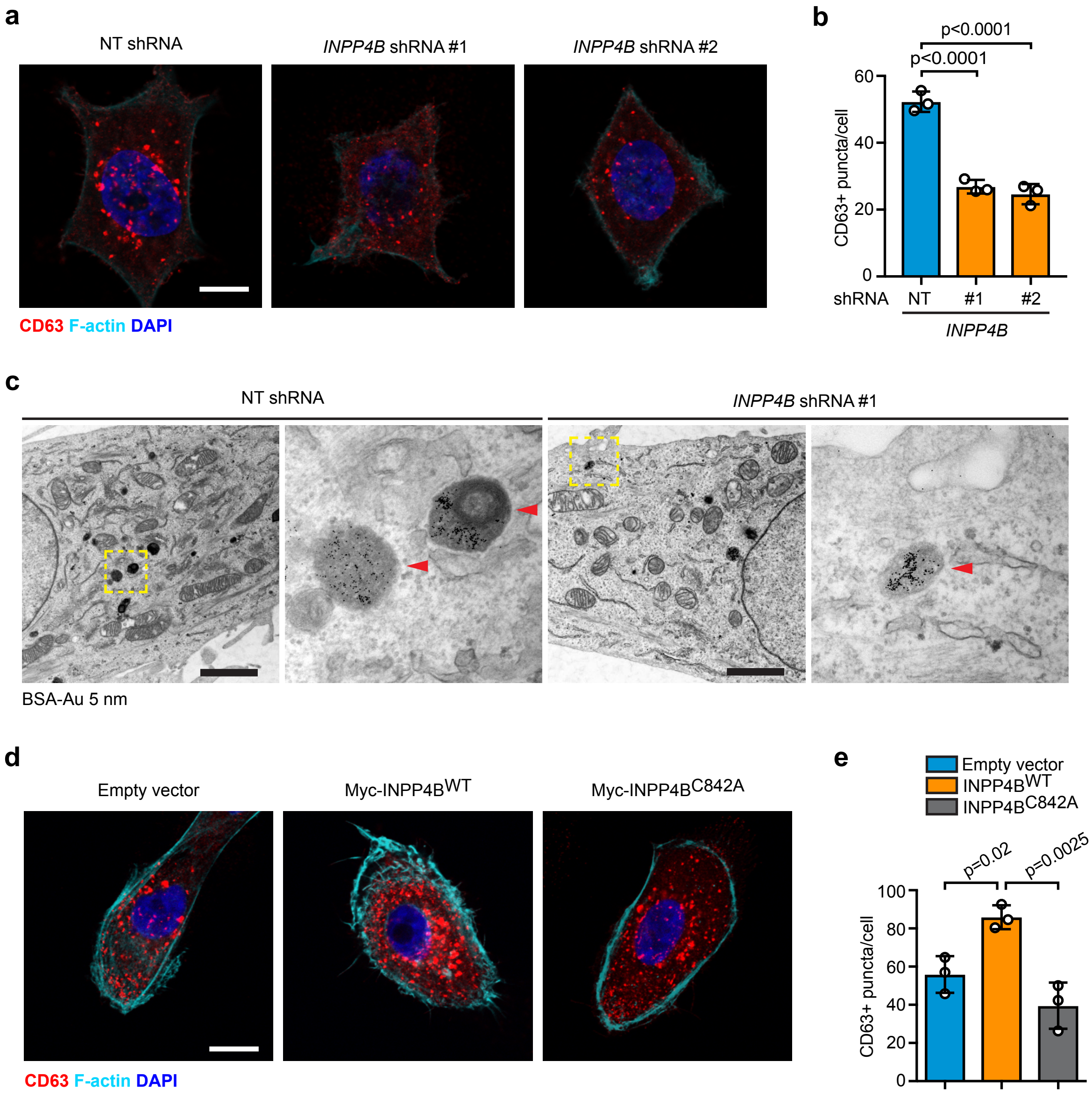

Supplementary Figure 4: *INPP4B* shRNA knockdown reduces late endosome formation.

(a, b) MCF-7 cells expressing NT, *INPP4B* #1 or *INPP4B* #2 shRNA were fixed and immunostained using CD63 antibodies, and co-stained with DAPI and phalloidin **(a)**. Data represent the number of CD63+ puncta per cell ± SD (n=3 experiments, >50 cells per experiment) **(b)**.

(c) MCF-7 cells expressing NT or *INPP4B* #1 shRNA were serum-starved for 1 hour, then growth media with BSA-gold (5 nm) was added for 3 hours. Cells were fixed and subjected to electron microscopy. Representative electron micrographs of lower and higher magnification are shown. Yellow boxes indicate area where higher magnification micrographs were captured.

(d, e) MCF-10A cells expressing Myc-INPP4B^{WT}, Myc-INPP4B^{C842A} or empty vector were fixed and immunostained using CD63 antibodies, and co-stained with DAPI and phalloidin **(d)**. Data represent the number of CD63+ puncta per cell ± SD (n=3 experiments, >50 cells per experiment) **(e)**.

Scale bar is 10 µm **(a, d)**, 2 µm **(c)**.

p values determined by one-way ANOVA with Tukey post hoc test are indicated in **(b, e)**.

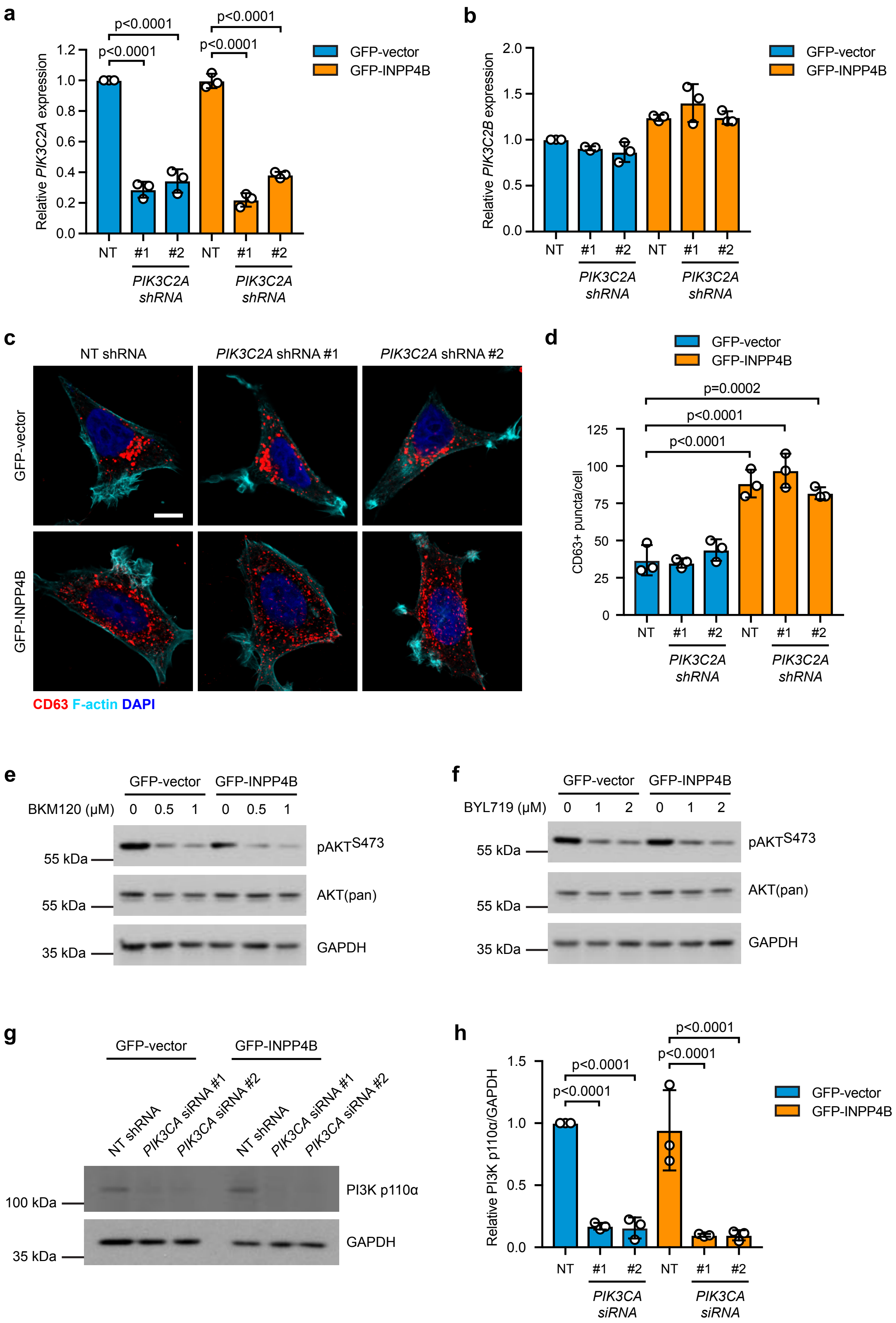
a
Relative PIK3C2A expression
1.2
1.0
0.8
0.6
0.4
0.2
0.0
p<0.0001
p<0.0001
p<0.0001
p<0.0001
NT
#1
#2
NT
#1
#2
PIK3C2A shRNA
PIK3C2A shRNA
GFP-vector
GFP-INPP4B
b
Relative PIK3C2B expression
2.0
1.5
1.0
0.5
0.0
NT
#1
#2
NT
#1
#2
PIK3C2A shRNA
PIK3C2A shRNA
GFP-vector
GFP-INPP4B
c
NT shRNA
PIK3C2A shRNA #1
PIK3C2A shRNA #2
GFP-vector
GFP-INPP4B
CD63 F-actin DAPI
d
GFP-vector
GFP-INPP4B
p=0.0002
p<0.0001
p<0.0001
CD63+ puncta/cell
125
100
75
50
25
0
NT
#1
#2
NT
#1
#2
PIK3C2A shRNA
PIK3C2A shRNA
e
GFP-vector
GFP-INPP4B
BKM120 (μM)
0
0.5
1
0
0.5
1
55 kDa
pAKT S473
55 kDa
AKT(pan)
35 kDa
GAPDH
f
GFP-vector
GFP-INPP4B
BYL719 (μM)
0
1
2
0
1
2
55 kDa
pAKT S473
55 kDa
AKT(pan)
35 kDa
GAPDH
g
GFP-vector
GFP-INPP4B
NT shRNA
PIK3CA siRNA #1
PIK3CA siRNA #2
NT shRNA
PIK3CA siRNA #1
PIK3CA siRNA #2
100 kDa
PI3K p110α
35 kDa
GAPDH
h
Relative PI3K p110α/GAPDH
1.5
1.0
0.5
0.0
p<0.0001
p<0.0001
p<0.0001
p<0.0001
NT
#1
#2
NT
#1
#2
PIK3CA siRNA
PIK3CA siRNA
GFP-vector
GFP-INPP4B

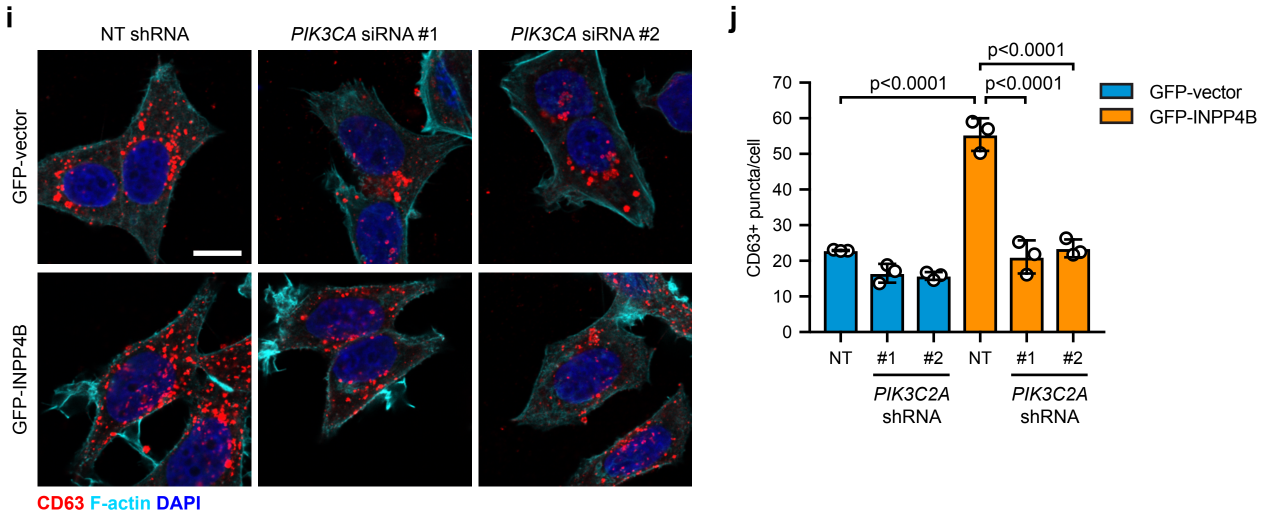

Supplementary Figure 5: *PIK3C2A* shRNA knockdown does not rescue the increased late endosome formation of INPP4B-overexpressing cells.

(a, b) MCF-7 cells expressing GFP-INPP4B or GFP-vector were transduced with lentiviral particles encoding NT, *PIK3C2A* shRNA #1 or *PIK3CA* shRNA #2. RNA was extracted and two-step quantitative RT-PCR was performed using primers for *PIK3C2A* **(a)** or *PIK3C2B* **(b)**. Expression was normalized to GAPDH. Expression was quantified from 3 independent experiments using the using the ΔΔCt method and expressed relative to GFP-vector;NT shRNA control cells (± SD) which were assigned an arbitrary value of 1.

(c, d) MCF-7 cells co-expressing GFP-INPP4B or GFP-vector, and NT, *PIK3C2A* shRNA #1 or *PIK3CA* shRNA #2, were fixed and immunostained using CD63 antibodies, and co-stained with DAPI and phalloidin **(c)**. Data represent the number of CD63+ puncta per cell ± SD (n=3 experiments, >50 cells per experiment) **(d)**.

(e, f) MCF-7 cells expressing GFP-INPP4B or GFP-vector were treated for 24 hours with BKM120 (0.5 or 1 μM) **(e)** or BYL719 (1 or 2 μM) **(f)**, or DMSO as a vehicle control. Cells were lysed and immunoblotted with phospho-AKT^{S473} or AKT(pan) antibodies, or GAPDH antibodies as a loading control.

(g, h) MCF-7 cells expressing GFP-INPP4B or GFP-vector were transfected with NT, *PIK3CA* #1 or *PIK3CA* #2 siRNA. After 48 hours, cells were lysed and immunoblotted with PI3K p110α antibodies, or GAPDH antibodies as a loading control **(g)**. Data represent expression of PI3K p110α relative to GAPDH expression ± SD (n=3 experiments) **(h)**.

(i, j) MCF-7 cells expressing GFP-INPP4B or GFP-vector were transfected with NT, *PIK3CA* #1 or *PIK3CA* #2 siRNA. After 48 hours, cells were fixed and immunostained using CD63 antibodies, and co-stained with DAPI and phalloidin **(i)**. Data represent the number of CD63+ puncta per cell ± SD (n=3 experiments, >40 cells per experiment) **(j)**.

Scale bar is 10 μm **(c, i)**.

p values determined by one-way ANOVA with Tukey post hoc test are indicated in **(a, d, h, j)**.

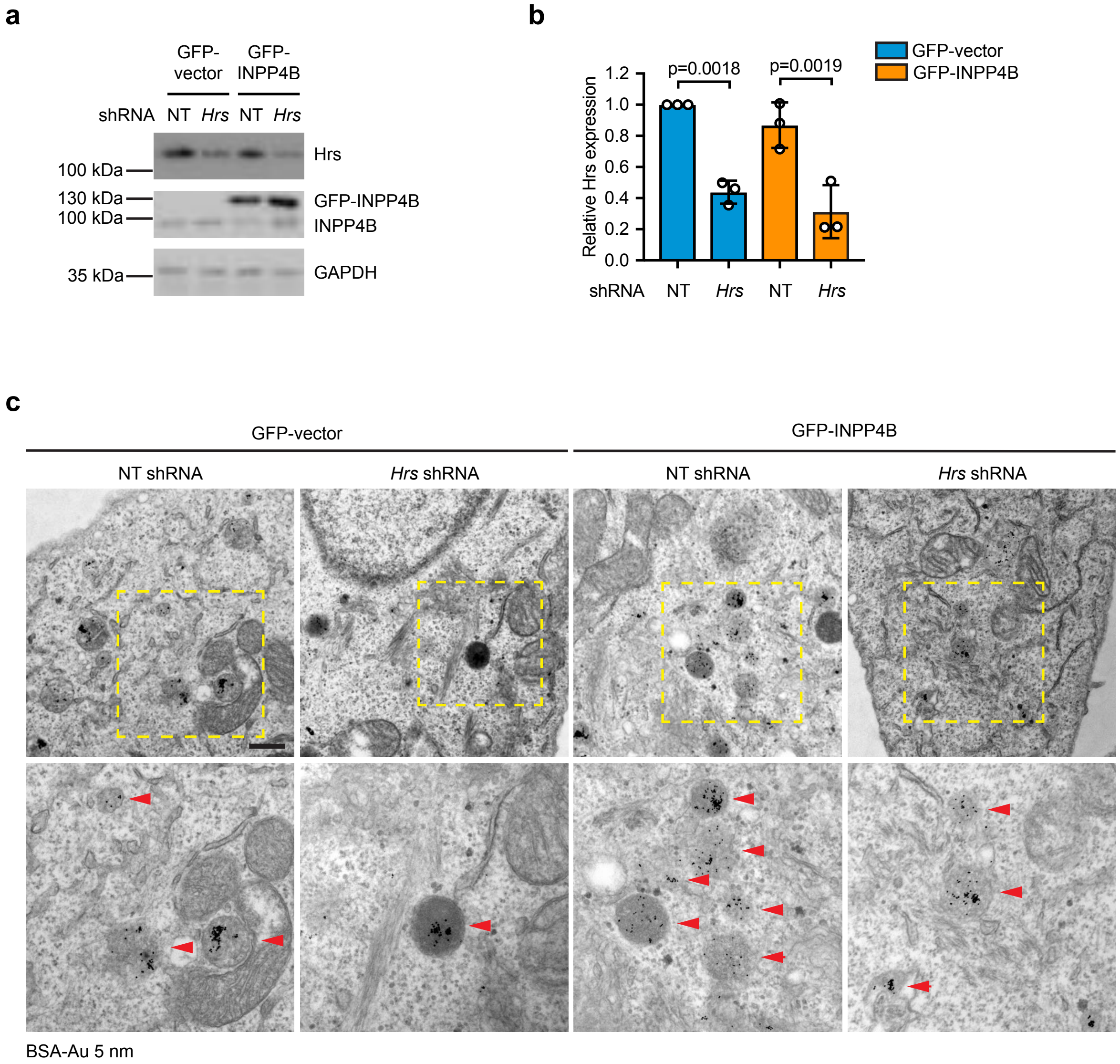

Supplementary Figure 6: Generation of stable *Hrs* shRNA knockdown in INPP4B-overexpressing breast cancer cells.

(a, b) MCF-7 cells expressing GFP-INPP4B or GFP-vector were transduced with lentiviral particles encoding NT or *Hrs* shRNA. Cells were lysed and subjected to immunoblotting with Hrs or INPP4B antibodies or GAPDH antibodies as a loading control **(a)**. Data represent expression of Hrs relative to GAPDH expression ± SD (n=3 experiments) **(b)**.

(c) MCF-7 cells co-expressing GFP-INPP4B or GFP-vector, and NT or *Hrs* shRNA, were serum-starved for 1 hour, then growth media with BSA-gold (5 nm) was added for 3 hours. Cells were fixed and subjected to electron microscopy. Representative electron micrographs of lower and higher magnification are shown. Yellow boxes indicate area where higher magnification micrographs were captured.

Scale bar is 500 nm **(c)**.

p values determined by one-way ANOVA with Tukey post hoc test are indicated in **(b)**.

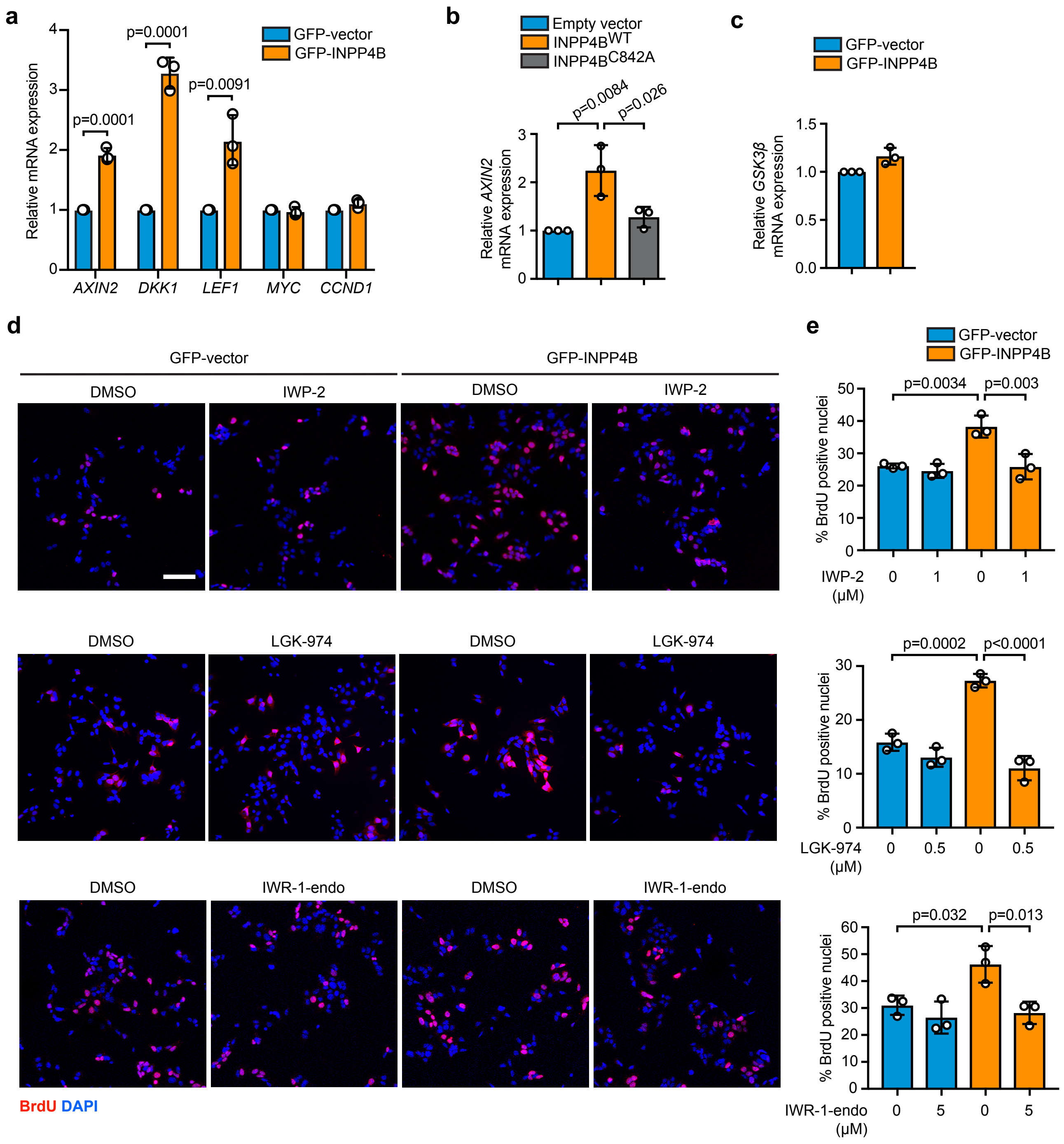

Supplementary Figure 7: Wnt inhibitors rescue INPP4B-mediated cell proliferation.

(a) RNA was extracted from T47D cells expressing GFP-INPP4B or GFP-vector, and two-step quantitative RT-PCR was performed using primers for *AXIN2*, *LEF1*, *DKK1*, *MYC* or *CCND1*. Expression was normalized to *GAPDH*. Expression was determined using the ΔΔCt method and expressed relative to GFP-vector control cells (± SD) which were assigned an arbitrary value of 1 (n=3 experiments).

(b) RNA was extracted from MCF-10A cells expressing INPP4B^{WT}, INPP4B^{C842A} or empty vector, and two-step quantitative RT-PCR was performed using primers for *AXIN2*. Expression was normalized to *GAPDH*. Expression was determined using the ΔΔCt method and expressed relative to empty vector control cells (± SD) which were assigned an arbitrary value of 1 (n=3 experiments).

(c) RNA was extracted from MCF-7 cells expressing GFP-INPP4B or GFP-vector, and two-step quantitative RT-PCR was performed using primers for *GSK3β*. Expression was normalized to *GAPDH*. Expression was determined the using the ΔΔCt method and expressed relative to GFP-vector control cells (± SD) which were assigned an arbitrary value of 1 (n=3 experiments).

(d, e) MCF-7 cells expressing GFP-INPP4B or GFP-vector were serum-starved overnight in the presence of 1 μM IWP-2, 0.5 μM LGK-974 or 5 μM IWR-1-endo, or DMSO as a vehicle control. Cells were incubated with BrdU overnight, then fixed and immunostained with BrdU antibodies and DAPI **(d)**. The mean percentage of BrdU-positive cells was quantified from 3 independent experiments (n>300 cells/experiment) **(e)**.

Scale bar is 100 μm **(d)**.

p values determined by two-tailed unpaired t test are indicated in (a), or by one-way ANOVA with Tukey post hoc test in **(b**, **e)**.

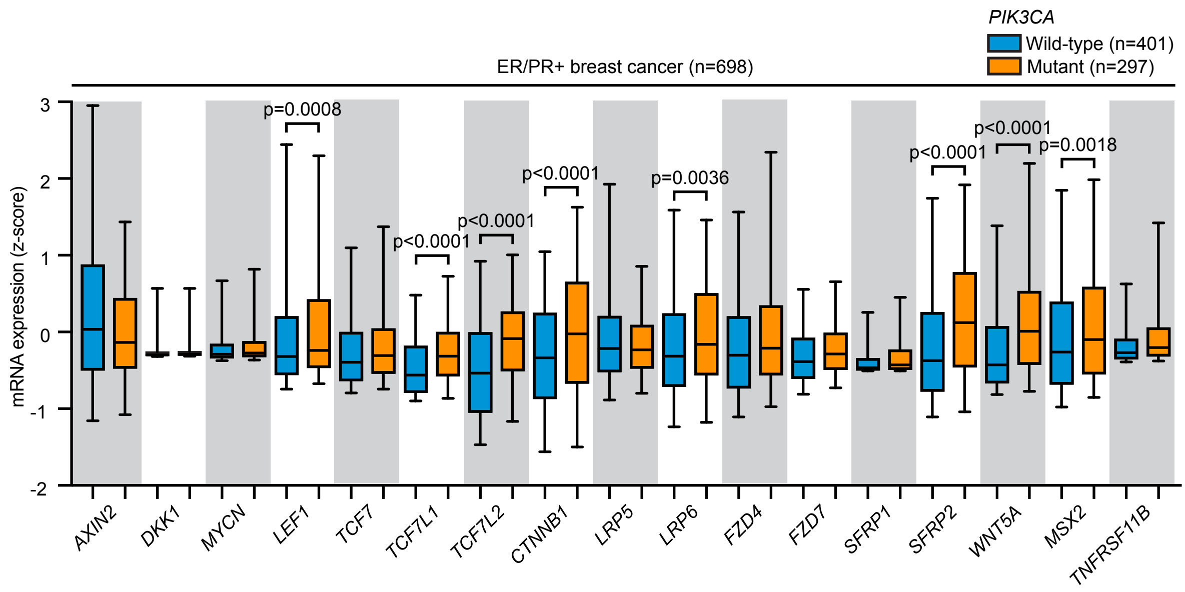

Supplementary Figure 8: *PIK3CA*-mutant ER/PR+ breast cancers exhibit increased Wnt/β-catenin gene expression.

Expression of a panel of 17 Wnt pathway genes was assessed in 698 ER/PR+ breast cancers from the TCGA cohort, and stratified by *PIK3CA*-mutation status. The centre line indicates the median, the lower bound of the box indicates the 25th percentile, the upper bound of the box represent the 75th percentile, the lower whisker extends from the 25th percentile to the 5th percentile, and the upper whisker extends from the 75th percentile to the 95th percentile.

p values determined by two-tailed unpaired Mann-Whitney are indicated.

Supplementary Table 1: Oligonucleotides

Human INPP4B (qRT-PCR)	OriGene	Cat # HP225789
Human ACTB (qRT-PCR)	OriGene	Cat # HP204660
Human AXIN2 (qRT-PCR)	Qiagen	Cat # QT00037639
Human LEF1 (qRT-PCR)	Qiagen	Cat # QT00021133
Human DKK1 (qRT-PCR)	Qiagen	Cat # QT00009093
Human MYC (qRT-PCR)	Qiagen	Cat # QT00201404
Human MYCN (qRT-PCR)	Qiagen	Cat # QT00035406
Human CCND1 (qRT-PCR)	Qiagen	Cat # QT00495285
Human GAPDH (qRT-PCR)	Qiagen	Cat # QT00079247
Human RRN18S (qRT-PCR)	Qiagen	Cat # QT00199367
HA forward primer (cloning) GACCATCCTCTAGACTGCCGGATCCATGGCTTCTAGCTATCCTTATGAC	Sigma	N/A
HA reverse primer (cloning) TAGAGGTCATGGGGTACCGAGCTCGAATTC	Sigma	N/A
Rab7 forward primer (cloning) TCGGTACCCCATGACCTCTAGGAAGAAAGTG	Sigma	N/A
Rab7 reverse primer (cloning) ATAAAATCTTTTATTTTATCGTCGACTCAGCAACTGCAGCTTTC	Sigma	N/A

Supplementary Table 2: Antibodies and dyes

Active-β-catenin (IB – 1:2000)	Merck	Cat # 05-665
AKT(pan) (IB - 1:1000)	Cell Signaling Technologies	Cat # 4691
β-catenin (IB – 1:2000)	BD Biosciences	Cat # 610153
CD63 (IF - 1:200, IEM – 1:50)	DSHB	Cat # H5C6
Cleaved caspase-3 (D175) (IF - 1:100)	Cell Signaling Technologies	Cat # 9661
EEA1 (IF - 1:200)	BD Biosciences	Cat # 610456
GAPDH (IB - 1:500,000)	ThermoFisher Scientific	Cat # AM4300
GFP (IB - 1:50,000)	Roche	Cat # 11814460001
GFP (biotin conjugated) (IEM - 1:600)	Rockland	Cat # 600-106-215
GM130 (IF - 1:500)	Sigma	Cat # G7295
GSK3β (IB - 1:1000)	Cell Signaling Technologies	Cat # 9315
GST (IF - 1:500)	Invitrogen	Cat # 71-7500
HA (IB – 1:1000, IF – 1:1600, IP – 1:200)	Cell Signaling Technologies	Cat # 3724
HA (IB – 1:5000)	Biolegend	Cat# MMS-101P
Hrs (IB - 1:1000)	Cell Signaling Technologies	Cat # 15087
INPP4B 3D5 (IB - 1:1000, IF – 1:50, IHC – 1:250)	[6]	N/A
Ki67 (IF – 1:200)	ThermoFisher Scientific	Cat # RM-9106-S1
LAMP1 (IF - 1:200)	DSHB	Cat # G1/139/5
Myc-tag (IB - 1:1000)	Cell Signaling Technologies	Cat # 2276
Phospho-AKT(S473) (IB - 1:1000)	Cell Signaling Technologies	Cat # 4058
Phospho-AKT(T308) (IB - 1:1000)	Cell Signaling Technologies	Cat # 2965
Phospho-SGK3(T320) (IB – 1:1000)	US Biological	Cat # S1010-85W8
PI(3,4)P_2 (IF – 1:200)	Echelon	Cat # Z-P034
PI3 Kinase p110α (IB – 1:2000)	Cell Signaling Technologies	Cat # 4249
PTEN (IB – 1:1000)	Cell Signaling Technologies	Cat # 9559
Rab7 (IB - 1:1000)	Cell Signaling Technologies	Cat # 9367

SGK3 (IB – 1:1000)	Cell Signaling Technologies	Cat # 8573
Anti-biotin (IEM - 1:10,000)	Rockland	Cat # 100-4198
Anti-mouse HRP-conjugated (IB - 1:10,000)	Millipore	Cat # AP308P
Anti-rabbit HRP-conjugated (IB - 1:10,000)	Millipore	Cat # AP307P
Anti-mouse IgG (H+L) (IEM - 1:1000)	Rockland	Cat # 610-4120
Anti-mouse IgG (H+L) Alexa-Fluor 488-conjugated (IF – 1:500)	Life Technologies	Cat # A-21200
Anti-mouse IgG (H+L) Alexa-Fluor 488-conjugated (IF – 1:500)	Life Technologies	Cat # A-11001
Anti-mouse IgG (H+L) Alexa-Fluor 555-conjugated (IF – 1:500)	Life Technologies	Cat # A-31570
Anti-mouse IgG1 Alexa-Fluor 555-conjugated (IF – 1:500)	Life Technologies	Cat # A-21127
Anti-mouse IgG1 Alexa-Fluor 647-conjugated (IF – 1:500)	Life Technologies	Cat # A-21240
Anti-mouse IgG2a Alexa-Fluor 488-conjugated (IF – 1:500)	Life Technologies	Cat # A-21131
Anti-rabbit IgG (H+L) Alexa-Fluor 488-conjugated (IF – 1:500)	Life Technologies	Cat # A-21206
Anti-rabbit IgG (H+L) Alexa-Fluor 555-conjugated (IF – 1:500)	Life Technologies	Cat # A-31572
DAPI (IF – 1 μg/mL)	Sigma	Cat # D9542
Propidium Iodide (IF - 1:1000)	Sigma	Cat # P4170
Phalloidin Texas Red™-X-conjugated (IF - 1:500)	Life Technologies	Cat # T7471
Phalloidin Alexa-Fluor 647-conjugated (IF - 1:500)	Life Technologies	Cat # A22287
Goat anti-mouse F(ab)’$_2$ fragment (IF - 20 μg/mL)	Jackson ImmunoResearch	Cat # 115-006-006
