# Supplementary material for: INPP4B promotes PI3Kα-dependent late endosome formation and Wnt/β-catenin signaling in breast cancer
Source: Nat Commun. 2021 May 25;12:3140. doi: 10.1038/s41467-021-23241-6 (PMC8149851; doi:10.1038/s41467-021-23241-6)
Supplement: Supplementary file 3 — Reporting Summary [file 41467_2021_23241_MOESM3_ESM.pdf]

nature research

Corresponding author(s): Christina Mitchell

Last updated by author(s): Mar 30, 2021

Reporting Summary

Nature Research wishes to improve the reproducibility of the work that we publish. This form provides structure for consistency and transparency in reporting. For further information on Nature Research policies, see our Editorial Policies and the Editorial Policy Checklist.

Statistics

For all statistical analyses, confirm that the following items are present in the figure legend, table legend, main text, or Methods section.

n/a	Confirmed	
☐	☒	The exact sample size (*n*) for each experimental group/condition, given as a discrete number and unit of measurement
☐	☒	A statement on whether measurements were taken from distinct samples or whether the same sample was measured repeatedly
☐	☒	The statistical test(s) used AND whether they are one- or two-sided<br>*Only common tests should be described solely by name; describe more complex techniques in the Methods section.*
☒	☐	A description of all covariates tested
☐	☒	A description of any assumptions or corrections, such as tests of normality and adjustment for multiple comparisons
☐	☒	A full description of the statistical parameters including central tendency (e.g. means) or other basic estimates (e.g. regression coefficient) AND variation (e.g. standard deviation) or associated estimates of uncertainty (e.g. confidence intervals)
☐	☒	For null hypothesis testing, the test statistic (e.g. *F*, *t*, *r*) with confidence intervals, effect sizes, degrees of freedom and *P* value noted<br>*Give P values as exact values whenever suitable.*
☒	☐	For Bayesian analysis, information on the choice of priors and Markov chain Monte Carlo settings
☒	☐	For hierarchical and complex designs, identification of the appropriate level for tests and full reporting of outcomes
☒	☐	Estimates of effect sizes (e.g. Cohen's *d*, Pearson's *r*), indicating how they were calculated

Our web collection on statistics for biologists contains articles on many of the points above.

Software and code

Policy information about availability of computer code

Data collection	Fluorescent microscopy images were taken using NIS-elements version 4.13 (Nikon) or LAS X version 3.5.6.21594 (Leica Microsystems).
Data analysis	Statistical analysis was performed using Prism version 7.0 (GraphPad). Fluorescent microscopy images were analysed using ImageJ version 2.0.0. qRT-PCR data was analysed using CFX Manager version 3.1 (Bio-Rad). Immunoblot data was analysed using ImageQuant (version 8.1.0.0) (GE Healthcare Life Sciences). Mass spectrometry data was analysed using MaxQuant version 1.5.2.8, Spectronaut version 8 (Biognosys) and Byonic version 3.1.0 (Protein Metrics). Functional annotation of the proteome was conducted using DAVID version 6.7. RNA profiling was analysed using nSolver version 3.0 (nanoString). Immunohistochemistry images were analysed using Aperio Imagescope version 12.4.0.5043 (Lecia Microsystems).

For manuscripts utilizing custom algorithms or software that are central to the research but not yet described in published literature, software must be made available to editors and reviewers. We strongly encourage code deposition in a community repository (e.g. GitHub). See the Nature Research guidelines for submitting code & software for further information.

Data

Policy information about availability of data

All manuscripts must include a data availability statement. This statement should provide the following information, where applicable:

- Accession codes, unique identifiers, or web links for publicly available datasets
- A list of figures that have associated raw data
- A description of any restrictions on data availability

The mass spectrometry proteomic .htrms files, .RAW files, and result files have been deposited to the Mass spectrometry Interactive Virtual Environment (MassIVE) consortium (https://massive.ucsd.edu/) with dataset identifier: MSV000085526. Data can also be found in the Proteome Xchange (http://proteomecentral.proteomexchange.org) under the Pride identifier: PXD019503. The DDA files were searched against the human UniProt fasta database (version

2015-08, 20,210 entries, https://ftp.uniprot.org/pub/databases/uniprot/previous_releases/release-2015_08/). Source data are provided with this paper. All other data that support the findings of this study are available from the corresponding author upon reasonable request.

Field-specific reporting

Please select the one below that is the best fit for your research. If you are not sure, read the appropriate sections before making your selection.

☒ Life sciences ☐ Behavioural & social sciences ☐ Ecological, evolutionary & environmental sciences

For a reference copy of the document with all sections, see nature.com/documents/nr-reporting-summary-flat.pdf

Life sciences study design

All studies must disclose on these points even when the disclosure is negative.

Sample size	No sample size calculation was performed. Mice numbers (8 per group) were determined by experience in xenografts study (Proc Natl Acad Sci U S A . 2010 Dec 21;107(51):22231-6). Immunoprecipitation-mass spectrometry analysis was performed using 2 independent replicates as this was sufficient to identify Rab7 binding, which was validated by immunoblotting. For electron microscopy experiments, at least 50 cell profiles were qualitatively assessed in a single experiment. All other experiments were repeated at least 3 times independently to ensure statistical significance of the results.
Data exclusions	Mice that died for unknown reasons were excluded from analysis. No other data were excluded.
Replication	Immunoprecipitation-mass spectrometry analysis was performed using 2 independent replicates. All other experiments were repeated at least 3 times independently. All attempts at replication were successful.
Randomization	Genetically modified cells in each experiments were derived from the same pool of parent cells. Cells were randomly assigned to treatment or control groups. Mice were randomly assigned to 8 per cage and each cage was used as an experimental group.
Blinding	For the immunohistochemistry, staining and analysis were performed blinded. No blinding in other experiments as the same investigator performed experiment and analyzed the data.

Reporting for specific materials, systems and methods

We require information from authors about some types of materials, experimental systems and methods used in many studies. Here, indicate whether each material, system or method listed is relevant to your study. If you are not sure if a list item applies to your research, read the appropriate section before selecting a response.

Materials & experimental systems

n/a	Involved in the study
☐	☒ Antibodies
☐	☒ Eukaryotic cell lines
☒	☐ Palaeontology and archaeology
☐	☒ Animals and other organisms
☒	☐ Human research participants
☒	☐ Clinical data
☒	☐ Dual use research of concern

Methods

n/a	Involved in the study
☒	☐ ChIP-seq
☒	☐ Flow cytometry
☒	☐ MRI-based neuroimaging

Antibodies

Antibodies used	Descriptions of antibodies used (including concentration, application, supplier name and catalog number) can be found in Supplementary Table 2: Antibodies and Dyes.
Validation	The INPP4B 3D5 antibody was validated by this lab as described in Proc Natl Acad Sci U S A . 2010 Dec 21;107(51):22231-6, Prostate . 2015 Jan;75(1):92-102, Blood . 2015 Apr 30;125(18):2815-24. CD63 and GFP (biotin conjugated) have been previously validated for immuno-electron microscopy (J Cell Biol . 2006 Jun 5;173(5):795-807. doi: 10.1083/jcb.200508165. Epub 2006 May 30, Sci Rep . 2021 Jan 13;11(1):899. doi: 10.1038/s41598-020-79637-9). All other antibodies area available commercially and the validation of each primary antibodies for the reactive species and applications provided by manufacturers as described:<br><br>Active-β-catenin (Merck, Cat # 05-665), Reactive species (human, mouse, rat), Applications (Western blotting, Immunohistochemistry, Immunocytochemistry, Flow Cytometry)<br>AKT(pan) (Cell Signaling Technologies, Cat # 4691), Reactive species (human, mouse, rat, monkey, D. melanogaster), Applications (Western blotting, Immunoprecipitation, Immunohistochemistry, Immunocytochemistry, Flow Cytometry)<br>β-catenin (BD Biosciences, Cat # 610153), Reactive species (human, mouse, rat, dog, chicken), Applications (Western blotting, Immunoprecipitation, Immunohistochemistry, Immunocytochemistry)<br>CD63 (DSHB, Cat # H5C6), Reactive species (human, porcine), Applications (Western blotting, Immunoprecipitation,

Immunocytochemistry, Flow Cytometry, ELISA)
Cleaved caspase-3 (Cell Signaling Technologies, Cat # 9661), Reactive species (human, mouse, rat, monkey), Applications (Western blotting, Immunoprecipitation, Immunohistochemistry, Immunocytochemistry, Flow Cytometry)
EEA1 (BD Biosciences, Cat # 610456), Reactive species (human, rat, dog, chicken), Applications (Western blotting, Immunocytochemistry)
GAPDH (ThermoFisher Scientific, Cat # AM4300), Reactive species (Amphibian, Dog, Chicken, Fish, Human, Mouse, Non-human primate, Rabbit, Rat), Applications (Western blotting, Immunohistochemistry, Immunocytochemistry)
GFP (Roche, Cat # 11814460001), Applications (Western blotting, Immunoprecipitation, Immunocytochemistry)
GSK3β (Cell Signaling technologies, Cat # 9315), Reactive species (human, mouse, rat, monkey), Applications (Western blotting, Immunoprecipitation, Immunocytochemistry)
GST (Invitrogen, Cat # 71-7500), Applications (Western blotting, Immunocytochemistry)
HA (Cell Signaling Technologies, Cat # 3724), Applications (Western blotting, Immunoprecipitation, Immunohistochemistry, Immunocytochemistry, Flow Cytometry)
HA (Biolegend, Cat# MMS-101P), Applications (Western blotting, Immunoprecipitation, Immunocytochemistry)
Hrs (Cell Signaling Technologies, Cat # 15087), Reactive species (human, mouse, monkey), Applications (Western blotting, Immunoprecipitation, Immunocytochemistry)
Ki67 (ThermoFisher Scientific, Cat # RM-9106-S1), Reactive species (human), Applications (Immunohistochemistry)
LAMP1 (DSHB, Cat # G1/139/5), Reactive species (human), Applications (Western blotting, Immunoprecipitation, Immunocytochemistry)
Myc-tag (Cell Signaling Technologies, Cat # 2276) Applications (Western blotting, Immunoprecipitation, Immunohistochemistry, Immunocytochemistry, Flow Cytometry)
Phospho-AKT(S473) (Cell Signaling Technologies, Cat # 4058), Reactive species (human, mouse, rat), Applications (Western blotting, Immunoprecipitation, Immunocytochemistry, Flow Cytometry)
Phospho-AKT(T308) (Cell Signaling Technologies, Cat # 2965), Reactive species (human, mouse, rat, hamster, monkey), Applications (Western blotting)
Phospho-SGK3(T320) (US Biological, Cat # S1010-85W8), Reactive species (human), Applications (Western blotting)
PI(3,4)P2 (Echelon, Cat # Z-P034), Applications (Immunofluorescence, ELISA, protein-lipid overlay)
PI3 Kinase p110α (Cell Signaling Technologies, Cat # 4249), Reactive species (human, mouse, rat, bovine), Applications (Western blotting, Immunoprecipitation)
PTEN (Cell Signaling Technologies, Cat # 9559), Reactive species (human, mouse, rat, monkey), Applications (Western blotting, Immunoprecipitation, Immunohistochemistry)
Rab7 (Cell Signaling Technologies, Cat # 9367), Reactive species (human, mouse, rat, monkey), Applications (Western blotting, Immunoprecipitation, Immunohistochemistry)
SGK3 (Cell Signaling Technologies, Cat # 8573), Reactive species (human, mouse, rat, monkey), Applications (Western blotting, Immunoprecipitation)

Eukaryotic cell lines

Policy information about cell lines

Cell line source(s)	MCF-7 (Cat # HTB-22), T47D (Cat # HTB-133), MCF-10A (Cat # CRL-10317) and HEK293T (Cat # CRL-3216) cells were purchased from ATCC.
Authentication	Cell line authentication was not performed.
Mycoplasma contamination	Cells were routinely tested to confirm the absence of mycoplasma contamination.
Commonly misidentified lines (See ICLAC register)	No commonly misidentified cell lines used.

Animals and other organisms

Policy information about studies involving animals; ARRIVE guidelines recommended for reporting animal research

Laboratory animals	7-8 week old female athymic BALB/c-Fox1nu/Asb mice obtained from Australian BioResources were used in this project. Mice were housed at 18-24°C with 40-70% humidity.
Wild animals	The study did not involve wild animals.
Field-collected samples	The study did not involve samples collected from the field.
Ethics oversight	All procedures involving mice were conducted in accordance with National Health and Medical Research Council (NHMRC) regulations on the use and care of experimental animals and the study protocol approved by the Monash University Animal Ethics Committee (project number MARP/2017/168).

Note that full information on the approval of the study protocol must also be provided in the manuscript.
