# Supplementary material for: INPP4B promotes PI3Kα-dependent late endosome formation and Wnt/β-catenin signaling in breast cancer
Source: Nat Commun. 2021 May 25;12:3140. doi: 10.1038/s41467-021-23241-6 (PMC8149851; doi:10.1038/s41467-021-23241-6)

Figure 2f

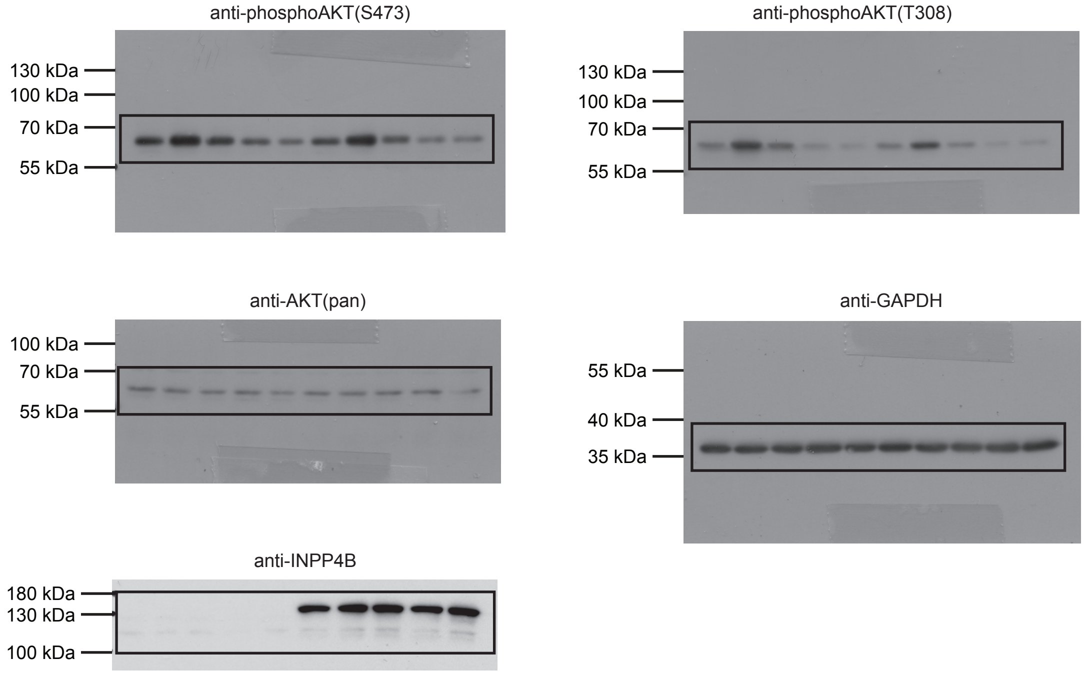

Figure 3c

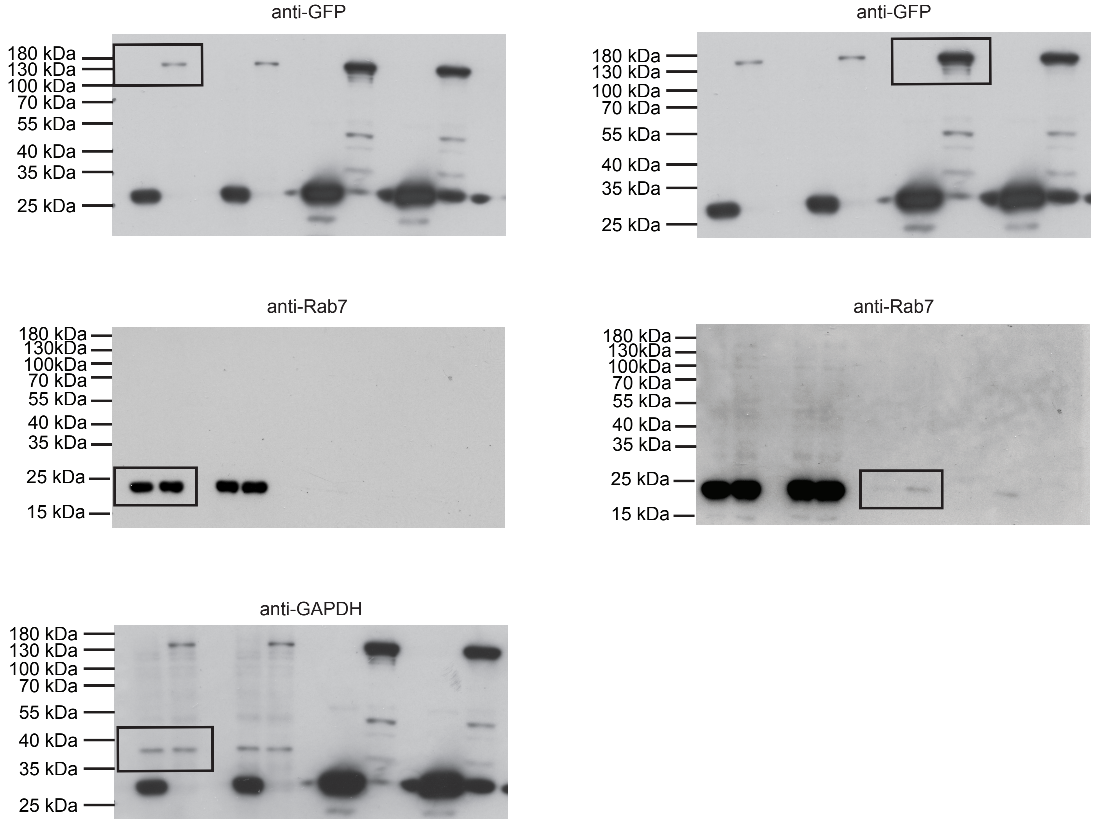

Figure 6d

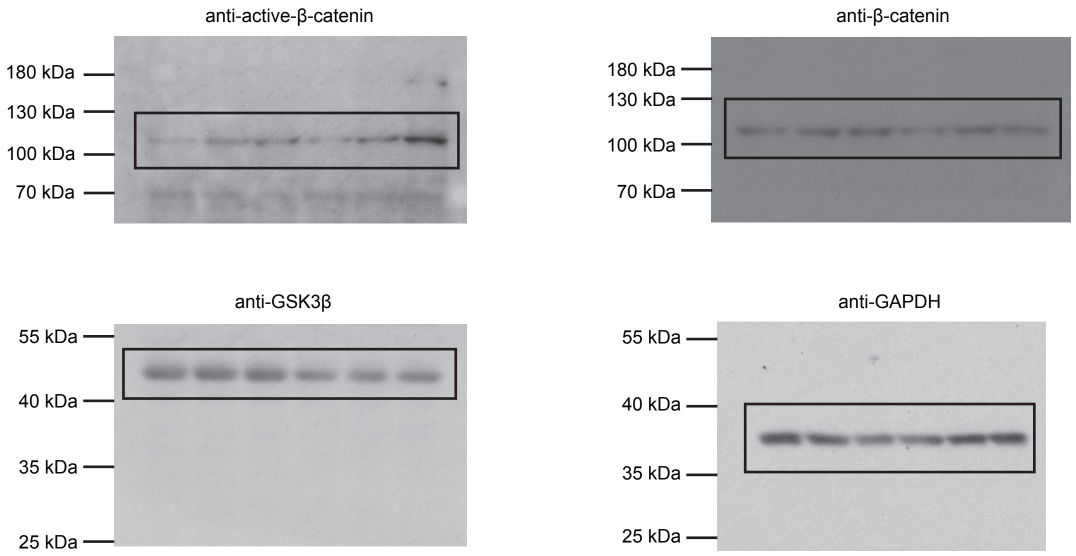

Figure 6f

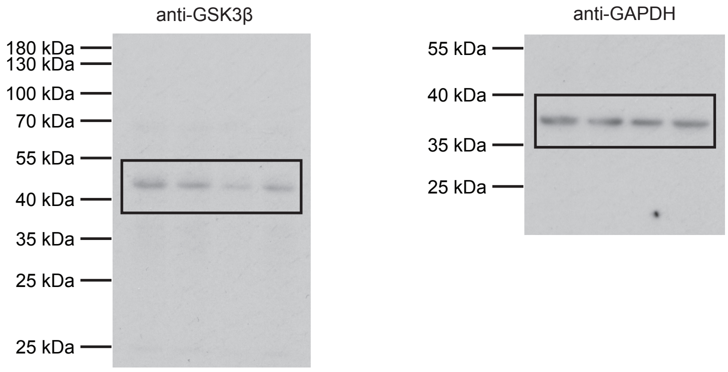

Figure 6h

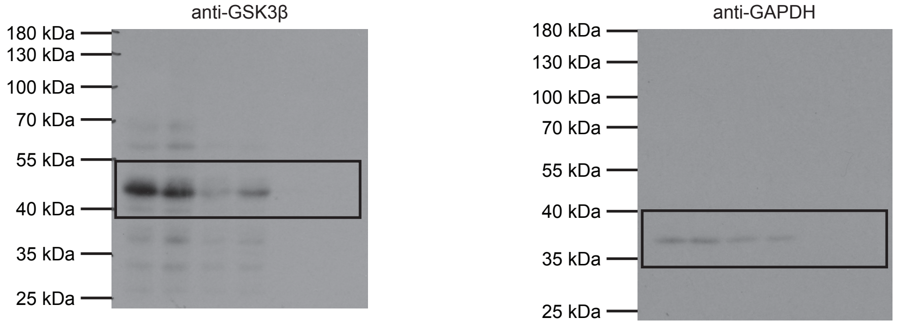

Supplementary Figure 2a

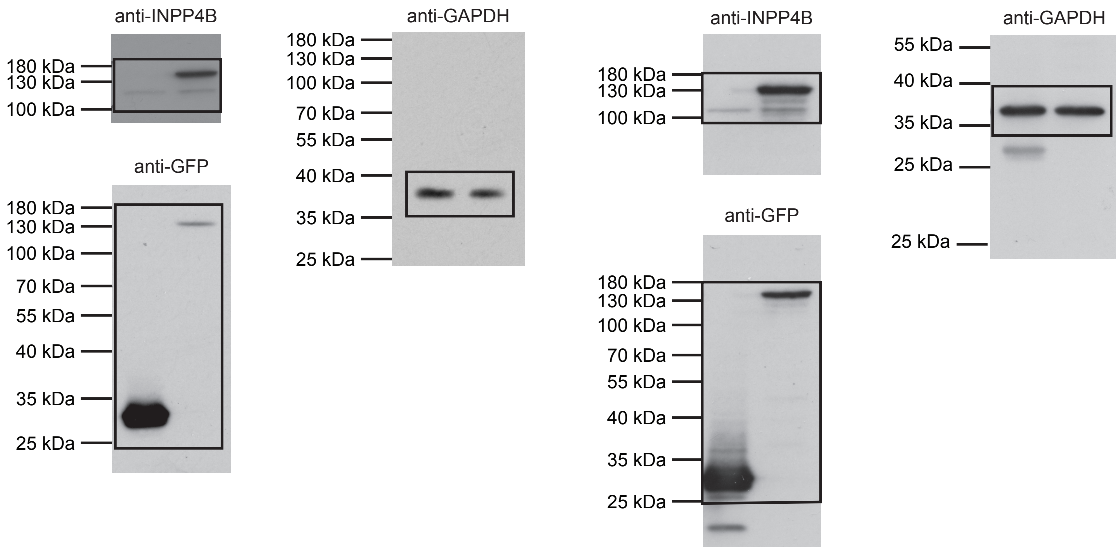

Supplementary Figure 2b

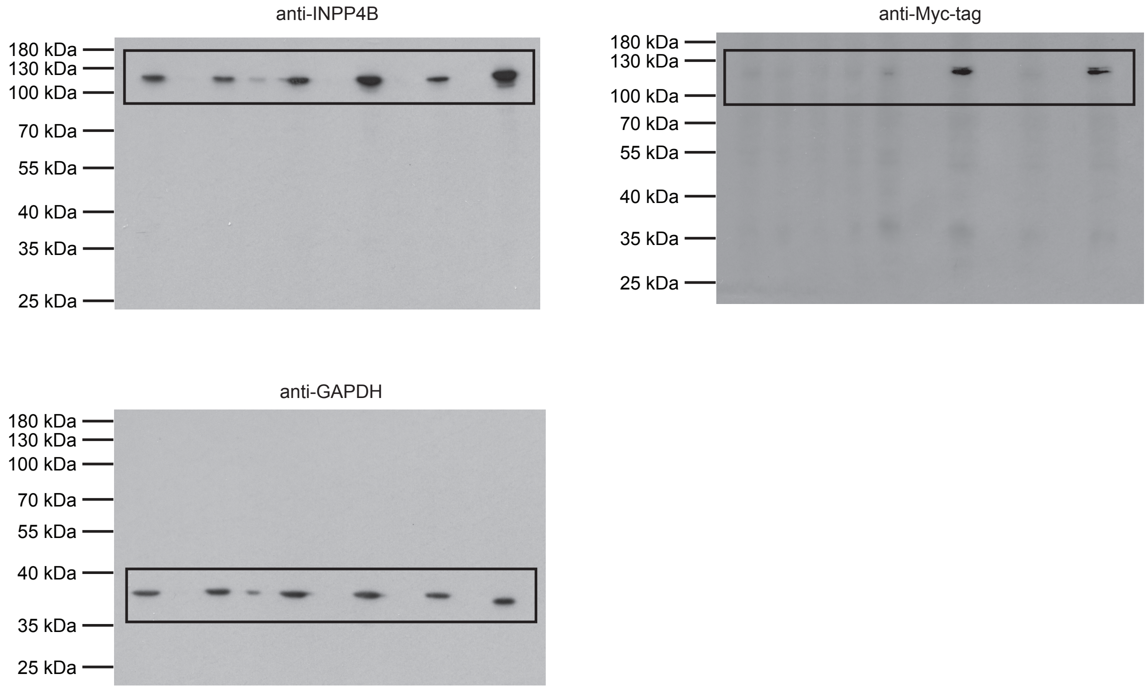

Supplementary Figure 2g

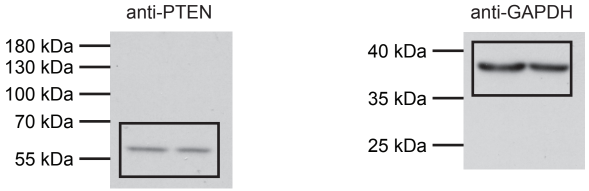

Supplementary Figure 2i

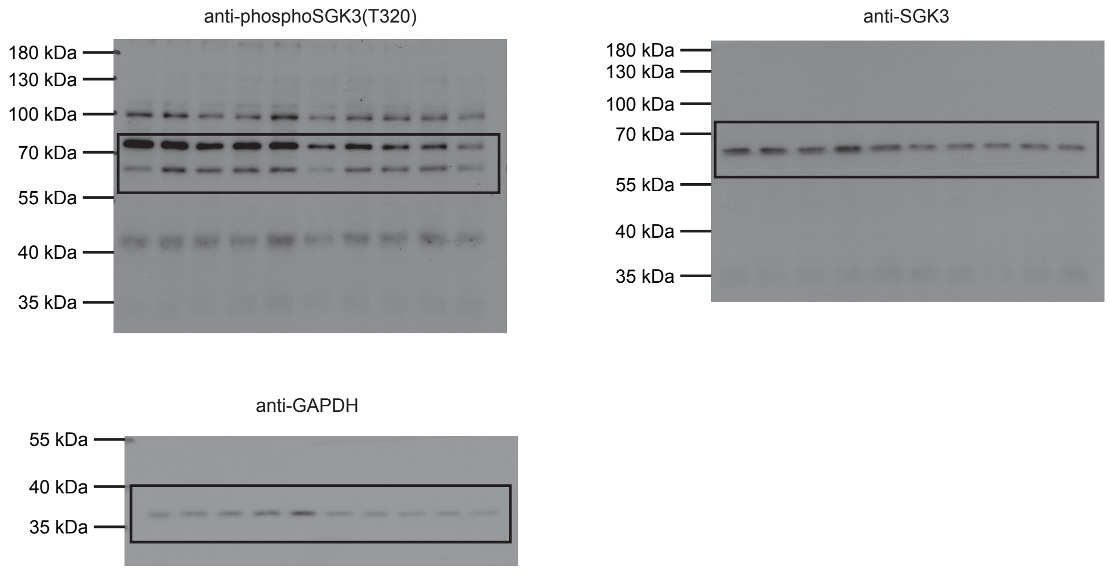

Supplementary Figure 2k

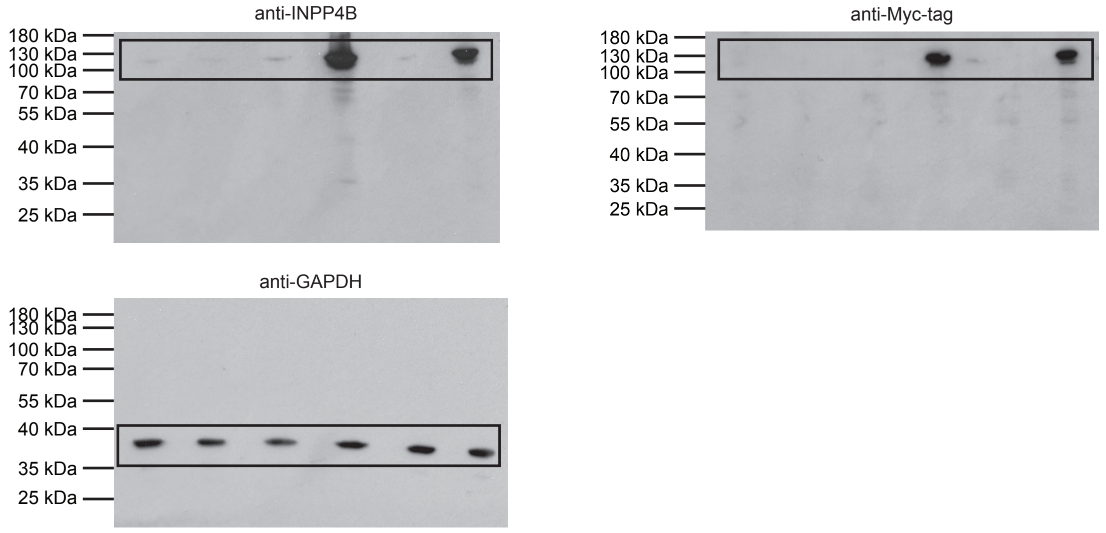

Supplementary Figure 2p

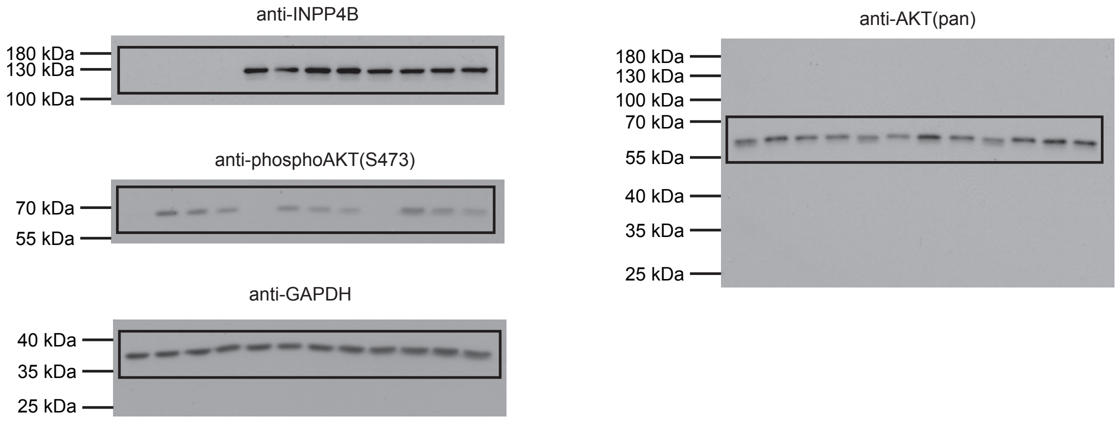

Supplementary Figure 3a

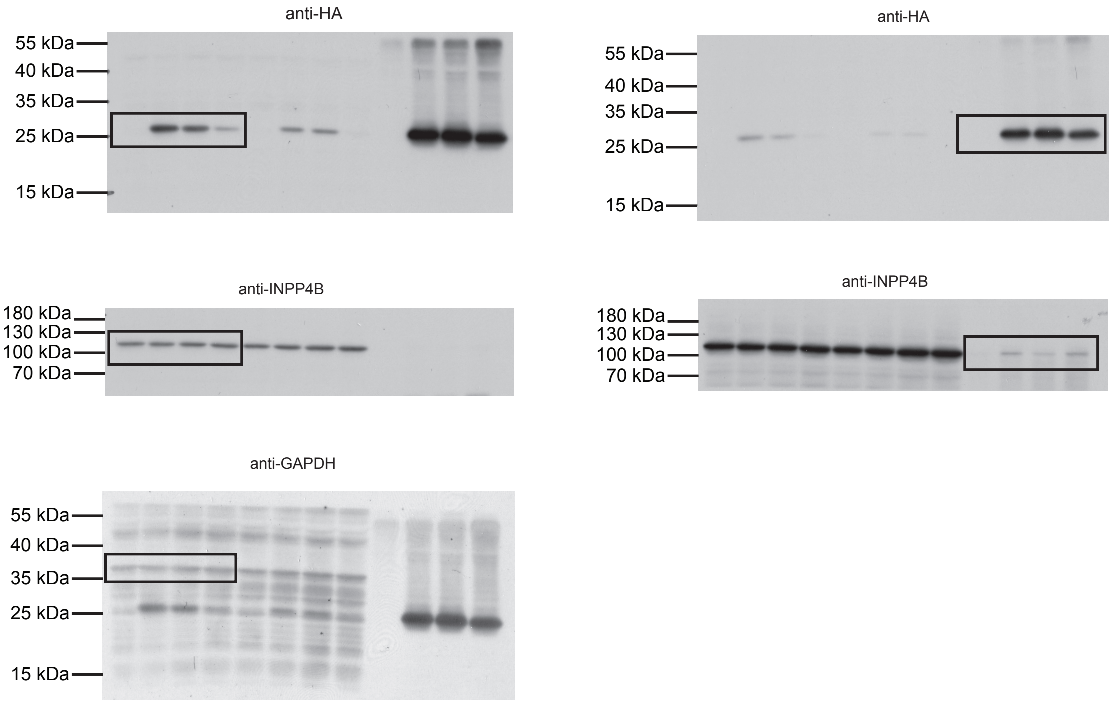

Supplementary Figure 3g

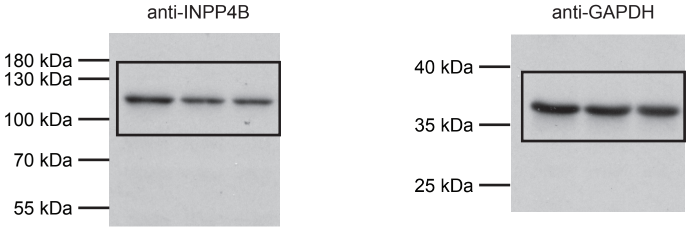

Supplementary Figure 5e

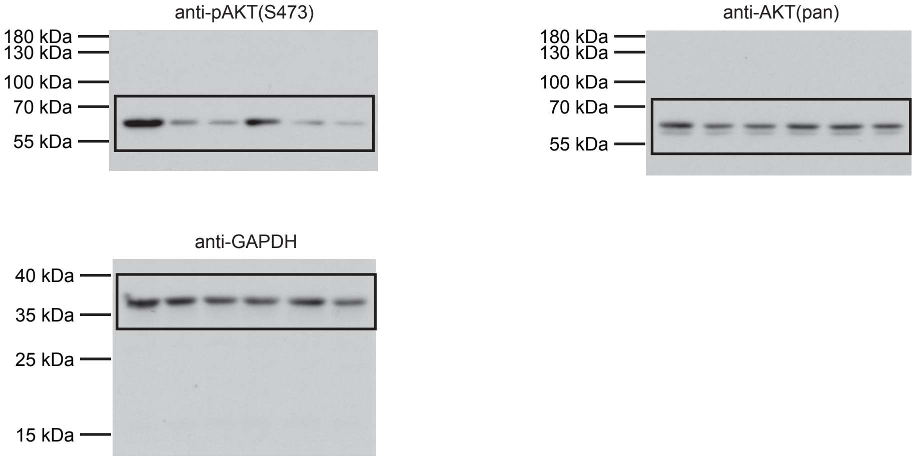

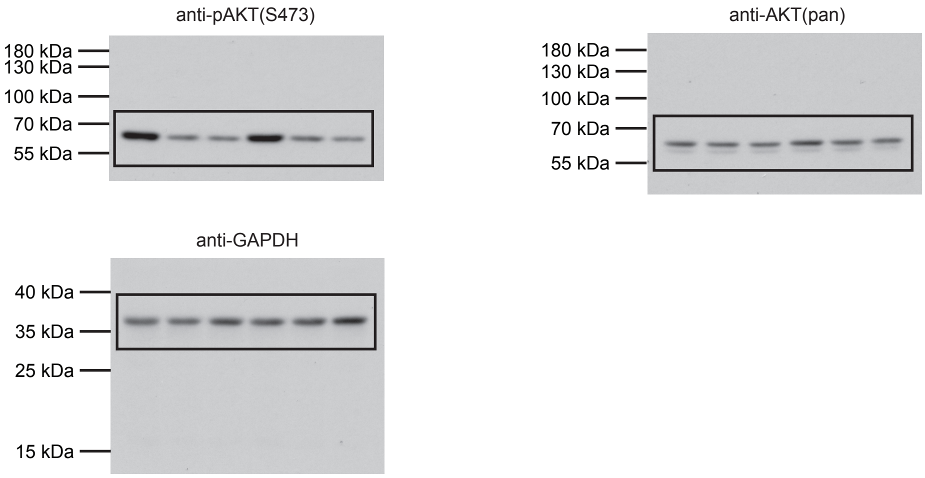

Supplementary Figure 5g

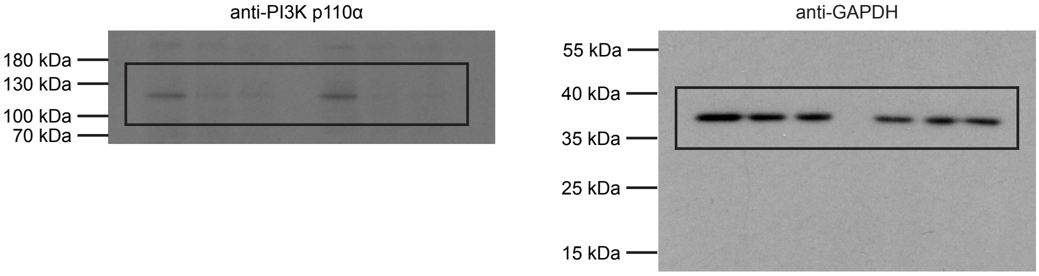

Supplementary Figure 6a

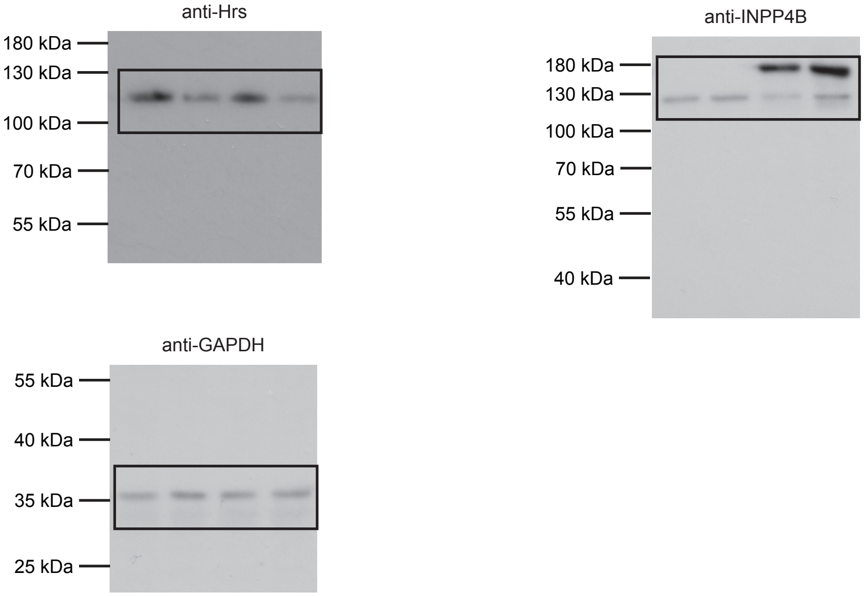

Supplement: Supplementary file 4 — Source Data [file 41467_2021_23241_MOESM4_ESM.zip › NCOMMS-20-40176-A Source data/Rodgers et al unprocessed blots.pdf]
